# The Catalytic Effect of Low Molecular Weight Acids on the Physicochemical and Dielectric Properties of Oil-Paper Insulation Systems

**DOI:** 10.3390/polym16182655

**Published:** 2024-09-20

**Authors:** Kakou D. Kouassi, Issouf Fofana, Yazid Hadjadj, Kouba M. Lucia Yapi

**Affiliations:** 1 Ufr-SSMT Laboratory of Technology, Université Félix Houphouet Boigny (UFHB), Abidjan 00225, Côte d’Ivoire; lorellmongoua@gmail.com; 2 Department of Applied Sciences (DSA), University of Quebec at Chicoutimi (UQAC), Saguenay, QC G7H 2B1, Canada; 3 Metrology Research Centre, National Research Council Canada (NRC), Ottawa, ON K1N 5A2, Canada

**Keywords:** carboxylic acids, paper insulation, oil insulation, acidity, dissipation factor

## Abstract

In most industrialized countries, power transformers built several decades ago are approaching the end of their operational lifespan. The ongoing energy transition, focused on developing 100% renewable energy sources and accelerating global transportation electrification, further exacerbates these assets. Combined with rising electricity demand, there is an increasing risk of critical transformers’ degradation acceleration. In this context, understanding the aging mechanisms of the insulation system inside these essential assets, which form the core of every energy network, becomes paramount for today’s managers and engineers responsible for their operations. The acids generated through oil oxidation can be classified into two categories: low molecular weight acids (LMAs), which are inherently more hydrophilic and consequently have a greater impact on the degradation rate of solid insulation through hydrolysis, and high molecular weight acids (HMAs), which do not significantly contribute to the degradation of paper insulation. This study specifically addresses the impact of acids generated through oil oxidation—focusing on LMAs. New oil samples were infused with different ratios of LMAs before impregnation. The impregnated paper samples underwent thermal aging at 115 °C. Different physicochemical and dielectric properties were investigated. The investigations revealed that oils blended with formic acid exhibited more adverse effects on the insulation system compared to other LMAs. This information is essential for industry professionals seeking to mitigate the risks associated with transformer degradation and extend the lifespan of these critical assets during the energy transition.

## 1. Introduction

Power transformers are highly expensive and essential components within the entire electrical energy transmission and distribution system. Often referred to as the “heart” of the grid, these machines are crucial. Failures in these transformers, primarily due to the degradation of their oil/paper insulation systems, can be extremely detrimental to the power grid [1,2,3,4,5,6,7]. Indeed, any failure of a power transformer disrupts the entire chain of the transmission and distribution of electrical energy. In these critical machines, dielectric materials (oil/paper insulation system) are placed between conductors/windings to ensure electrical isolation. While the power transformer operates, chemical reactions occur within the oil/paper insulation system, leading to the formation of degradation products that accelerate aging. Throughout this aging process, acids are generated as a result of these chemical reactions. The oxidation of the transformer’s oil and the hydrolysis of the paper insulation material produce carboxylic acids (CAs) [8]. CAs formed in oil are classified into two groups: low molecular weight (LMA) and high molecular weight (HMA).

Low molecular weight carboxylic acids (LMAs) consist of acetic, formic, and levulinic acids [9]. LMAs have an affinity with cellulose (paper) and water because of their ability to bond with the hydroxyl group (-OH). They chemically react to yield a proton (H+), as described by the acid–base theory of Brønsted-Lowry [8,9]. These carboxylic acids (LMAs) in the oil/paper insulation system thus accelerate its degradation [9]. As degraded insulating materials, paper and oil could achieve a higher dielectric loss and a lower interfacial tension, respectively. The increase in the dielectric loss of oil/paper insulation is one of the warning indices of the condition of the transformer. 

With the ever-increasing demand for energy, the power transmission voltages and the performance requirements for the insulation of power transformers are increasing. The traditional mineral oils used inside these important machines are thus facing a serious challenge to meet those requirements. Several studies are being carried out to modify the fluid to achieve better electrical properties (such as breakdown strength, permittivity, conductivity, etc.). A particular emphasis is laid on the effect of nanoparticles on the electrical property enhancement of nano-modified insulating oil [10]. Meanwhile, in the course of searching for insulating materials with high performance, a large effort is also devoted to the development of or improvement in efficient methods to monitor the degradation of the insulation systems and to prevent unexpected failure during operation [10].

The interfacial tension and the loss factor are among the commonly used indicators for the insulation’s health condition assessment [11]. These parameters increase with aging by-products such as moisture and LMAs [11,12]. 

This contribution investigates the correlations between the degradation indicators: the IFT, dissipation factor, and moisture/LMA content.

## 2. Materials and Methods

### 2.1. Oil and Paper Sample Pretreatment

The mineral oil utilized in this study is Nytro Polaris GX, which was degassed and dehumidified over 48 h. Kraft paper samples with dimensions of 8 × 8 cm^2^ were prepared. The paper and pressboard were dried in a vacuum oven at 110 °C for 24 h. Typically, manufacturers establish a mass ratio of paper to oil based on the unit’s rating. In our experiments, the mass ratio between cellulose insulation and oil was 20:1. A total of 2000 mL of oil, corresponding to 1812 g in mass, was used. This quantity of oil corresponds to a combined mass of 90.60 g for the paper and pressboard. The paper was dried for 20 min to facilitate the oil’s penetration into the paper and pressboard pores.

### 2.2. Preparation of Oil with Different Ratios of LMA

Oil samples, each with a volume of 2 L, were infused with three types of LMA at two different concentrations: 0.2 mg KOH/g and 0.4 mg KOH/g. These preparations are detailed in Table 1. The paper samples were then impregnated with these pre-formulated acidic oil samples and subjected to accelerated open-beaker thermal aging at 115 °C up to 2000 h.

### 2.3. Determination of Interfacial Tension (IFT) of Aged Oil Samples

The interfacial tension (IFT) is the force in dynes/cm required to tear a platinum ring at the interface between the oil and the distilled water. The IFT is influenced by the presence of the polar compounds which are precursors to sludge [13,14]. This parameter was assessed according to ASTM D971.

### 2.4. Determination of Moisture in Oil Insulation

The moisture content in a transformer measured in parts per million (ppm) is a critical factor affecting its insulation system and overall performance. Moisture degrades the insulation materials, reducing their dielectric strength and accelerating aging processes. At high operating temperatures, moisture can vaporize, forming bubbles. These bubbles can cause partial discharges and insulation breakdowns. To determine the water content, the ASTM D1533 standard was used with the help of an 831 Karl Fisher coulometric titrator (Metrohm: Herisau, Switzerland).

### 2.5. Determination of Dissipation Factor of Oil/Paper Insulation

The dissipation factor (also known as the loss tangent or tan δ) is a key parameter used to assess the condition of transformer insulation, including oil and paper insulation. It indicates the dielectric losses in the insulation system and provides insight into its aging, health, and electrical performance. The dissipation factor was measured using the IDA 200 instrument (Insulation Diagnostic Analyzer (Megger: Norristown, PA, USA)) [15] with a testing cell for liquids, following the ASTM D924 standard. This method involves applying an AC voltage to the oil sample and measuring the resulting dielectric loss at a line frequency of 60 Hz and 90 °C.

## 3. Results

### 3.1. Oil Samples

Oil samples with different acid values were thermally aged at 115 °C in a convection oven for 2000 h, with samples taken every 500 h for analysis. Sampling at regular 500 h intervals ensures consistency in data collection, which is crucial for comparing the aging characteristics of the insulating oil over time. This approach allows for a systematic analysis of the degradation process. While it is acknowledged that significant changes can occur in the pre-aging period, the 500 h interval was chosen to balance the need for detailed observation with practical constraints. This interval is sufficient to capture the key trends and significant changes in the oil’s properties without overwhelming the study with excessive data points. Table 2 presents a series of photographs depicting various oil samples to which different concentrations of carboxylic acids—formic, acetic, and levulinic—have been added, specifically 0.20 mg KOH/g and 0.40 mg KOH/g, illustrating the impact of thermal aging on the oil. A change in oil color, from light to dark brown, is observed with increasing aging time and concentration of carboxylic acids in the oil samples. This change typically signifies significant degradation of the oil [13]. After 500 h (approximately 21 days) of aging at 115 °C, it was observed that all oil samples, despite their varying acidity levels, changed color from light to shades such as pale yellow, yellow, bright yellow, and amber. Specifically, the oil sample containing formic acid at a concentration of 0.40 mg KOH/g appeared darker compared with the other samples at the same time interval. This condition suggests that the oil sample holding formic acid at 0.40 mg KOH/g is more degraded compared with the oil sample containing formic acid at 0.20 mg KOH/g. At 1000 h (about 42 days) of thermal aging, all of the oil samples with their different acidity levels can be seen.

### 3.2. Interfacial Tension (IFT) of Aged Oil Samples

**Figure 1:**
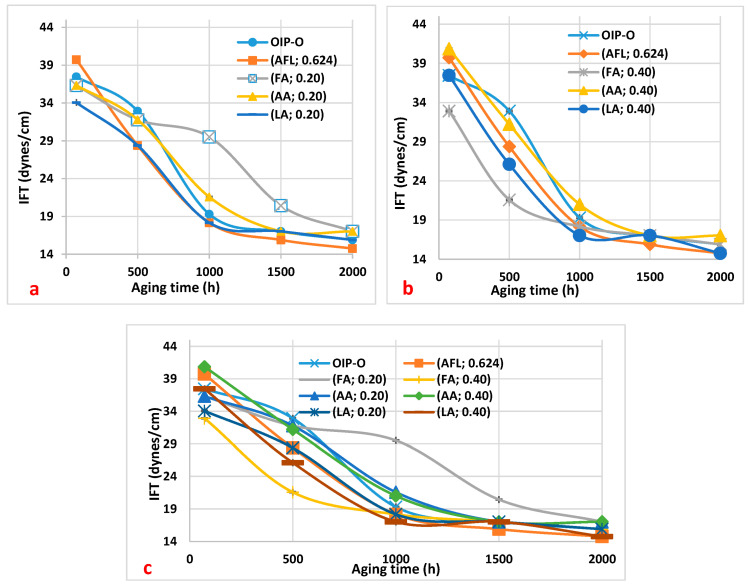
Interfacial tension (IFT) variations in oil samples over aging duration, affected by carboxylic acids. (**a**) IFT response with 0.20 mg KOH/g of formic acid (FA), acetic acid (AA), and levulinic acid (LA), their collective mixture (AFL) at 0.20 mg KOH/g, and baseline oil with paper (OIP). (**b**) IFT response with 0.40 mg KOH/g of FA, AA, and LA, AFL mixture at 0.20 mg KOH/g, and baseline OIP. (**c**) Synthesized comparison of IFT results from (**a**,**b**) for analytical clarity.

In Figure 1, the variation in interfacial tension (IFT) with aging time is displayed, alongside the concentrations of low molecular weight acids (LMAs). The figure includes acetic acid (AA), formic acid (FA), and levulinic acid (LA), as well as a composite mixture of these acids (AFL). These are compared to a baseline of neutral oil-impregnated paper (OIP), illustrating the distinct effects that each acid concentration has on IFT during the aging process.

Figure 1 displays the trends in the interfacial tension (IFT) of the oil over a thermal aging period ranging from 0 to 2000 h, with a focus on the effects of three carboxylic acids at concentrations of 0.20 mg KOH/g and 0.40 mg KOH/g. The observed decline in IFT is indicative of the oil’s degradation, correlating with an increase in polar contaminants like carboxylic acids (resulting from the oil’s oxidation process) [13,14].

Specifically, Figure 1a details the IFT for oil samples at 0.20 mg KOH/g acidity. Notably, the oil containing formic acid demonstrates a consistently higher IFT compared to oil samples with acetic and levulinic acids at identical concentrations, particularly between 500 and 2000 h of aging. An increase in the IFT of the oil with formic acid indicates that formic acid is more prevalent or has a greater influence compared to other LMAs present in the oil. Therefore, a significant change in interfacial tension may suggest a higher concentration or activity of formic acid relative to other acids in the oil.

Conversely, Figure 1b illustrates that the IFT for the oil sample with 0.40 mg KOH/g of formic acid is lower up to 1000 h of aging, signifying more pronounced degradation relative to the other samples. Finally, in Figure 1c, the curve of the sample of oil with added formic acid at 0.20 mg KOH/g is above the other curves. This means that this oil sample is less degraded than the other ones.

This result corroborates the findings regarding acidity levels and the outcomes shown in Table 2 for the oil samples. Therefore, in line with the instance of the oil sample enriched with 0.20 mg KOH/g of formic acid, where the oil exhibits less degradation, the higher interfacial tension observed in this sample also suggests lower degradation of the oil.

### 3.3. Moisture in Oil Insulation

**Figure 2:**
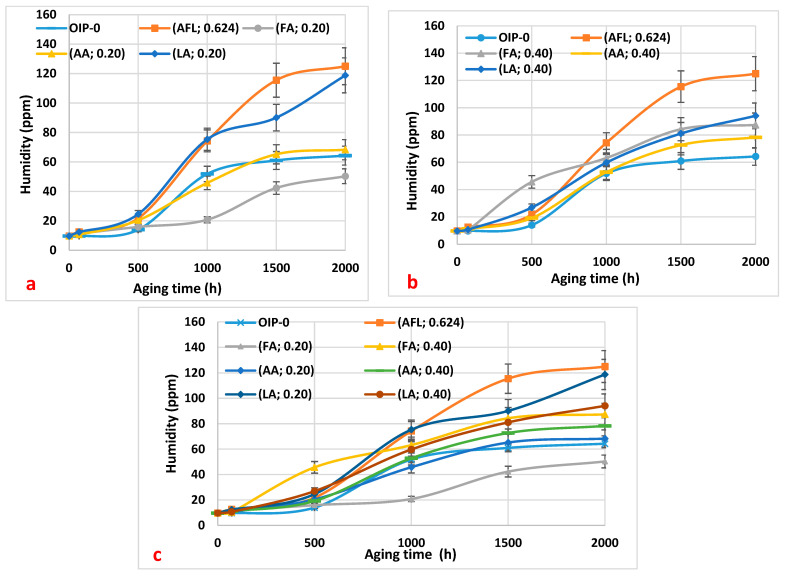
The variation in the moisture of the oil samples as a function of thermal aging duration: (**a**) 0.20 mg KOH/g of the three acids (formic, acetic, and levulinic), a mixture of the three acids at 0.20 mg KOH/g, and virgin oil within paper (HNAP); (**b**) 0.40 mg KOH/g of the three acids (formic, acetic, and levulinic), a mixture of the three acids at 0.20 mg KOH/g (mixture), and virgin oil within paper (HNAP); (**c**) a summary of the results from (**a**,**b**) for a comparison.

The initial water content of each oil type was recorded before the aging process began. Throughout the aging period, changes in water content were monitored at regular intervals. Figure 2a–c illustrates the trends in moisture content observed in the oil samples during thermal aging at 115 °C. The data show that oil containing higher contents of acids tended to absorb more moisture over time, likely due to the hygroscopic nature of the acids. This increase in water content can accelerate the degradation process, as water acts as a catalyst for hydrolysis reactions. Across all samples infused with different concentrations of acetic, formic, and levulinic acids, there was a notable increase in moisture levels. In Figure 2a, which illustrates the variation in the oil’s humidity for all carboxylic acids with acidities of 0.20 mg KOH/g, we observe that the oil sample containing formic acid (0.20 mg KOH/g) exhibits lower humidity compared to the other acids at the same concentration during thermal aging between 500 and 2000 h. This decrease in the oil’s humidity with formic acid suggests that the oil undergoes less degradation over time with this acid. Consequently, the amount of water resulting from moisture migration from paper to oil is reduced. Additionally, the degradation of the oil due to acid hydrolysis produces less water. In Figure 1b, it can be seen that all of the oil samples degrade at a similar rate. Figure 1c, however, shows that the oil sample with 0.20 mg KOH/g of formic acid is less degraded, as indicated by its lower humidity level.

Let us recall that moisture migration from paper to oil is influenced by the operating temperature of power transformers, which necessitates a moisture equilibrium between the oil and paper components. In practical terms, this dynamic facilitates the reciprocal transfer of moisture during transformer operation. Our experimental findings support this phenomenon, demonstrating a decrease in paper moisture concurrent with an increase in oil moisture [15].

### 3.4. Dissipation Factor of Oil/Paper Insulation

#### 3.4.1. Loss Factor of Oil

**Figure 3:**
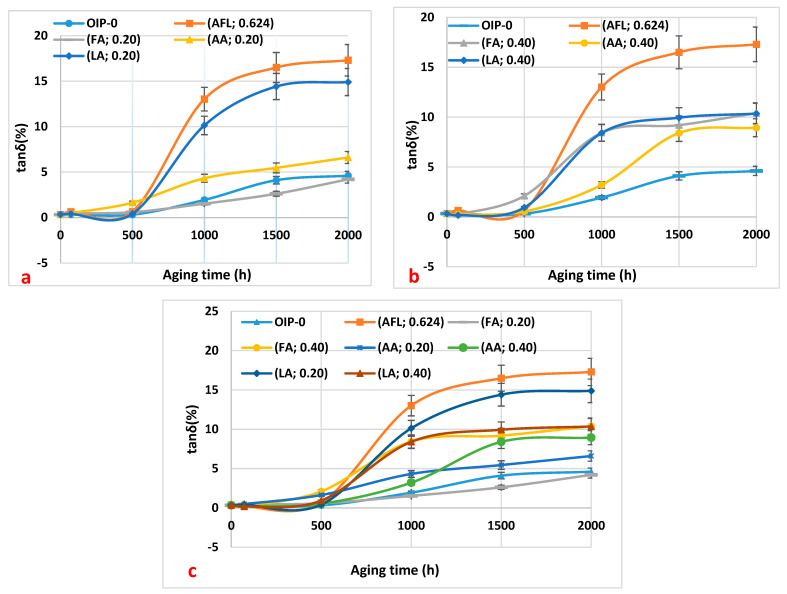
The variation in the loss factor of the oil samples as a function of thermal aging duration: (**a**) 0.20 mg KOH/g of the three acids (formic, acetic, and levulinic), a mixture of the three acids at 0.20 mg KOH/g, and virgin oil within paper (HNAP); (**b**) 0.40 mg KOH/g of the three acids (formic, acetic, and levulinic), a mixture of the three acids at 0.20 mg KOH/g (mixture), and virgin oil within paper (HNAP); (**c**) a summary of the results from (**a**,**b**) for a comparison.

Figure 3a–c shows the results of the loss factor of the oil samples with respect to thermal aging duration.

The dissipation factor of all oil samples increases with the duration of thermal aging, up to 2000 h. This increase is attributed to the presence of moisture and oil-soluble polar contaminants formed during oxidation degradation [11]. Specifically, the dissipation factor of the oil sample containing 0.20 mg KOH/g of formic acid shows a very slight increase during aging. This minimal change is due to the low concentration of formic acid in the oil. This trend aligns with the consistently low acidity value of 0.20 mg KOH/g of formic acid observed during aging (Figure 3a). Furthermore, the dissipation factor of the other samples exhibits slightly higher values because these acids are less soluble in oil. 

Carboxylic acids are polar compounds, and their presence in the oil leads to an increase in the dissipation factor. Since the oil sample with 0.20 mg KOH/g of formic acid has lower acidity during aging, its dissipation factor remains lower throughout the aging process. Furthermore, Figure 3a–c indicates that the dissipation factor of the oil sample containing the acid mixture is higher. This could be attributed to the higher moisture content observed from 1000 to 2000 h (Figure 2a–c).

#### 3.4.2. Loss Factor of Paper

**Figure 4:**
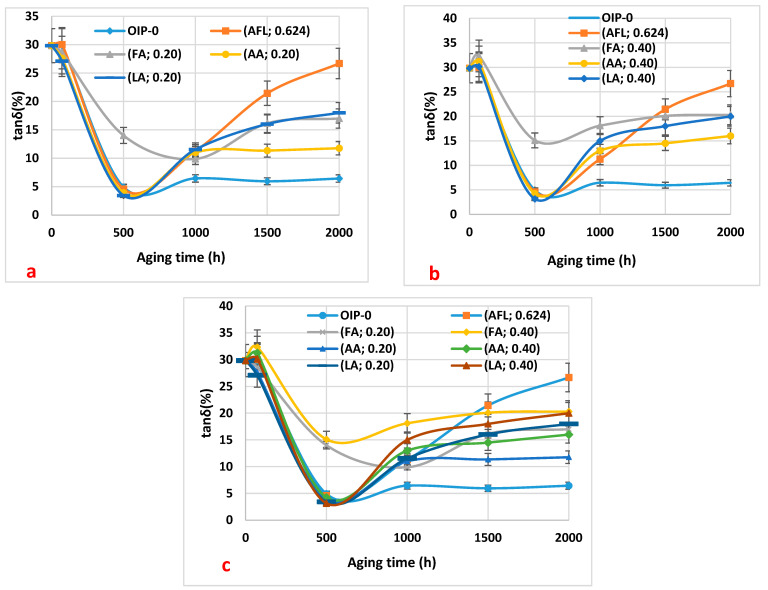
The loss factor of the paper samples as a function of thermal aging duration: (**a**) 0.20 mg KOH/g of the three acids (formic, acetic, and levulinic), a mixture of the three acids at 0.20 mg KOH/g, and virgin oil within paper (HNAP); (**b**) 0.40 mg KOH/g of the three acids (formic, acetic, and levulinic), a mixture of the three acids at 0.20 mg KOH/g (mixture), and virgin oil within paper (HNAP); (**c**) a summary of the results from (**a**,**b**) for a comparison.

Figure 4a–c show the results of the loss factor of the impregnated paper samples concerning thermal aging duration at 115 °C. A very steep decrease in the loss factor for all paper samples from 0 h to 500 h of thermal aging at 115 °C was observed. After 500 h of aging, the dissipation factor starts increasing again for the paper samples, but the rate of that increase depends on the acid and its concentration in the paper sample.

The decrease in the loss factor for all paper samples for the first few hours of thermal aging may be due to the acid hydrolysis reaction of cellulose, which leads to the consumption of water molecules during aging. It could also be [16] a process of mass transfer of water resulting from an imbalance where moisture passes from paper to oil by means of diffusion phenomena during continuous aging at high temperatures [15].

Both situations lead to a decrease in moisture in the paper, which in turn results in a decrease in the loss factor of the paper samples from 0 to 500 h of thermal aging. The loss factor of the impregnated paper samples with formic acid remains higher after 500 h of aging. This may be due to a larger amount of formic acid in the paper. The low acidity of formic acid in the oil sample means that the formic acid has migrated into the paper samples. Therefore, there has been a mass transfer of formic acid from oil into paper by means of diffusion phenomena.

From 500 to 2000 h of aging, an increase in the loss factor of all samples is observed. This increase could result from low molecular weight carboxylic acids being absorbed by the paper [11,12]. However, a very low loss factor of the paper is observed for virgin oil during aging.

Therefore, the reduction in moisture content in the paper and the absorption of low molecular weight carboxylic acids (LMAs) contribute to the peak in the dissipation factor observed at 500 h of aging.

## 4. Discussion

Low molecular weight acids can have several effects on paper insulation in transformers:

- Corrosion and Degradation: Low molecular weight acids, such as acetic acid, formic acid, and others, can be by-products of cellulose degradation in paper insulation. These acids can corrode metallic components and accelerate the degradation of paper itself. This degradation can weaken the structural integrity of paper insulation, leading to reduced dielectric strength and an increased risk of failure.
- pH Balance: Paper insulation in transformers typically functions well within a specific pH range. Low molecular weight acids can alter the pH of the insulation material, potentially leading to acidic conditions. These acidic conditions can further accelerate the breakdown of cellulose fibers in the paper, compromising the mechanical and electrical properties of the insulation system.
- Hygroscopic Nature: Some low molecular weight acids are hygroscopic, meaning they tend to absorb moisture from the surrounding environment. In transformer insulation, this can lead to increased moisture content in the paper. Moisture combined with acids can further promote chemical reactions that degrade cellulose and reduce the overall insulation effectiveness.
- Electrical Properties: The presence of acids can alter the dielectric properties of the insulation material. Acidic conditions may lead to partial discharges or corona formation within the transformer, which can further degrade the insulation over time and increase the risk of electrical faults.

Based on these studies, it is evident that current diagnostic methods for assessing the condition of insulation oil which rely solely on total acid number (which is a measure of the number of acidic substances in the oil including HMA) do not offer a comprehensive view of transformer health. 

Vorbeck et al. (1960) demonstrated that by modifying Craig and Murty’s method, it is feasible to separate and identify lower molecular weight fatty acids using the same chromatography column employed for higher molecular weight acids [17]. Gas Chromatography can be used to separate and quantify individual acids, including LMAs, based on their retention times. Recently, Yan et al. [18] proposed a water extraction-based measurement technique of LMAs in mineral oil and synthetic ester.

The investigations clearly show that regular monitoring of acidity levels and moisture content in transformer insulation is crucial to assess the health of the insulation. 

Since the condition of dielectric paper directly influences the service life of power transformers, new standard measurement techniques capable of distinguishing between low molecular weight acids would be valuable for monitoring the state of solid insulation systems. Meanwhile, maintenance practices may include drying processes or chemical treatments to neutralize acids and restore optimal conditions for paper insulation.

## 5. Conclusions

Low molecular weight acids (LMAs) play a significant role in insulating oil and paper aging. LMAs, such as formic and levulinic acids, are by-products of the degradation process and can accelerate further degradation through acid hydrolysis. This self-catalyzing reaction can lead to a faster breakdown of paper insulation, impacting the overall aging of the equipment. This holds critical importance given the current state of aging power transformers, especially in industrialized countries where many were built decades ago and are now nearing the end of their operational lifespan. Understanding the aging mechanisms of the insulation systems within these essential assets is of utmost importance for managers and engineers tasked with ensuring their reliability. Transformer failure can result in costly outages and risks to the stability of energy systems, particularly as electricity demand continues to rise.

In this study, the moisture content, acidity, and dissipation factor of oil and oil-impregnated paper samples were monitored while undergoing thermal aging at 115 °C. The presence of LMAs was also monitored to understand their influence on the aging process. The data collected reflect the combined effects of thermal aging and acid-induced degradation, providing a comprehensive view of the insulating oil’s performance over time. Various low molecular weight carboxylic acids were added to the oil, and these samples were used to impregnate the paper. The results indicate that formic acid, at both concentrations (0.20 and 0.40 mg KOH/g), is significantly absorbed by the oil-impregnated paper samples. This absorption leads to the deterioration of the dielectric properties of paper insulation, evidenced by an increase in the dissipation factor after 500 h of thermal aging.

Additionally, the oil sample containing 0.40 mg KOH/g of formic acid exhibits the lowest interfacial tension (IFT) value. Conversely, the oil with a lower quantity of formic acid (0.20 mg KOH/g) shows less degradation, characterized by a lower moisture content and dissipation factor, and higher IFT.

Ultimately, formic acid mostly contributes to the deterioration of the oil/paper insulation system in power transformers. This information is important for industry professionals who typically rely on the oil’s total acid number (TAN) to assess the condition of transformers and make informed decisions regarding maintenance and lifespan extension.

## Figures and Tables

**Table 1:** Quantitative summary of acid additions.

Acid Concentration	0.20 mg KOH/g	0.40 mg KOH/g
Virgin oil		
Oil with acetic acid	X	X
Oil with formic acid	X	X
Oil with levulinic acid	X	X
Oil with acid mixture (acetic, formic, and levulinic)	X	

**Table 2:** Photograph of thermally aged oils.

Carboxylic Acids		HNAP	AA (Acetic Acid)		FA (Formic Acid)		LA (Levulinic Acid)		Mixture
		0.0036	0.20	0.40	0.20	0.40	0.20	0.40	0.624
**Temps**									
00 h		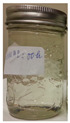	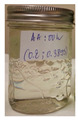	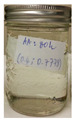	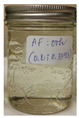	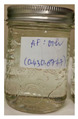	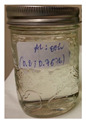	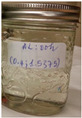	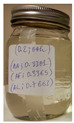
500 h		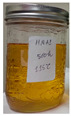	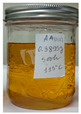	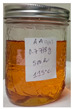	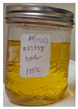	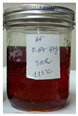	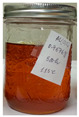	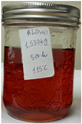	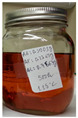
1000 h		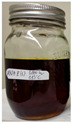	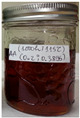	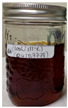	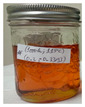	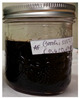	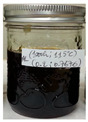	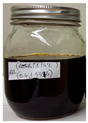	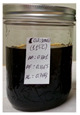
1500 h		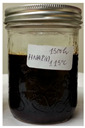	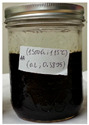	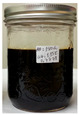	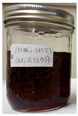	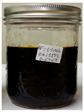	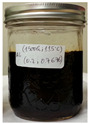	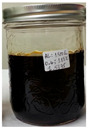	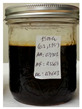
2000 h		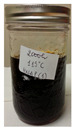	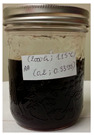	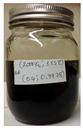	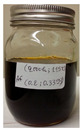	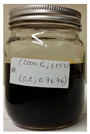	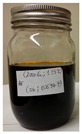	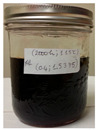	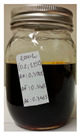

## Data Availability

The raw data were generated at UQAC. The derived data supporting the findings of this study are available from the corresponding author [I.F.] on request.
